# Phospholipase D1 deficiency in mice causes nonalcoholic fatty liver disease via an autophagy defect

**DOI:** 10.1038/srep39170

**Published:** 2016-12-15

**Authors:** Jang Ho Hur, Shi-Young Park, Claudia Dall’Armi, Jae Sung Lee, Gilbert Di Paolo, Hui-Young Lee, Mee-Sup Yoon, Do Sik Min, Cheol Soo Choi

**Affiliations:** 1 Department of Molecular Medicine, Lee Gil Ya Cancer and Diabetes Institute, Gachon University School of Medicine, Incheon 406-840, Korea; 2 Korea Mouse Metabolic Phenotyping Center (KMMPC), Lee Gil Ya Cancer and Diabetes Institute, Gachon University School of Medicine, Incheon 406-840, Korea; 3 Department of Pathology and Cell Biology, Columbia University Medical Center, New York, NY 10032, United States of America; 4 Department of Molecular Biology, College of Natural Science, Pusan National University, Busan 609-735, Korea; 5 Endocrinology, Internal Medicine, Gachon University Gil Medical Center, Incheon 405-760, Korea

## Abstract

Nonalcoholic fatty liver disease (NAFLD) is characterized by the accumulation of triglycerides (TG) as lipid droplets in the liver. Although lipid-metabolizing enzymes are considered important in NAFLD, the involvement of phospholipase D1 (PLD1) has not yet been studied. Here, we show that the genetic ablation of PLD1 in mice induces NAFLD due to an autophagy defect. PLD1 expression was decreased in high-fat diet-induced NAFLD. Subsequently, PLD1 deficiency led to an increase in hepatic TGs and liver weight. Autophagic flux was blocked in *Pld1*^−/−^ hepatocytes, with decreased β-oxidation rate, reduced oxidation-related gene expression, and swollen mitochondria. The dynamics of autophagy was restored by treatment with the PLD product, phosphatidic acid (PA) or adenoviral PLD1 expression in *Pld1*^−/−^ hepatocytes, confirming that lysosomal PA produced by PLD1 regulates autophagy. Notably, PLD1 expression in *Pld1*^−/−^ liver significantly reduced hepatic lipid accumulation, compared with *Pld1*^−/−^ liver. Thus, PLD1 plays an important role in hepatic steatosis via the regulation of autophagy.

Nonalcoholic fatty liver disease (NAFLD) is a chronic liver disease and a major hepatic health problem worldwide^1^. NAFLD is characterized by hepatic macrovesicular steatosis without any obvious cause of secondary fat accumulation, such as significant alcohol consumption^2^. It is strongly connected to metabolic syndromes such as obesity, hypertension, dyslipidemia, and insulin resistance^3^. Despite its prevalence and importance, the underlying mechanisms of NAFLD induction are poorly characterized.

One of the primary factors for hepatic steatosis is an imbalance of the lipid flux in the liver^4^. Various factors result in lipid accumulation in the liver, e.g., increased lipolysis in adipose tissues and/or high dietary fat intake, increased de novo hepatic lipid synthesis, a decrease in fat oxidation, and decreased hepatic very low-density lipoprotein (VLDL) secretion^4^,^5^. Furthermore, an autophagy defect in the liver induces hepatic steatosis, which is accompanied by a reduced β-oxidation rate^6^. Several genetic studies have shown that deficiencies in fatty acid-metabolizing enzymes result in NAFLD, and elevated levels of diacylglycerol (DAG) are an indicator of NAFLD-induced insulin resistance. These findings underscore the importance of lipid-metabolizing enzymes in NAFLD and subsequent insulin resistance^7^,^8^. Despite the importance of DAG in NAFLD and insulin resistance, the roles of other DAG-convertible lipids in NAFLD are not clear.

DAG is converted to phosphatidic acid (PA) by DAG kinases and the reverse reaction is catalyzed by PA phosphatases^9^. PA is a lipid second messenger involved in membrane transport and several important signaling cascades including mammalian target of rapamycin (mTOR). In addition, several PA species may be involved in insulin signaling^10^,^11^. Di-16:0 PA dampens protein kinase B (Akt) phosphorylation in insulin-stimulated hepatocytes by disrupting the interaction between mTOR and rapamycin-insensitive companion of mTOR (rictor)^10^. Additionally, 16:0/18:1 PA and 18:1/20:4 PA enhance hepatic glucose production in AGPAT2^−/−^ mice by elevating the expression of glucose-6-phosphatase and phosphoenolpyruvate carboxykinase, resulting in hyperglycemia^11^. However, the functions of PA species from different enzymatic reactions in hepatic steatosis have not been demonstrated.

PLD1 hydrolyzes phosphatidylcholine (PC) to produce PA, which contains fatty acid chains with one or two unsaturated bonds^12^. The PA species produced by PLD1 (primarily 16:0/18:1 PA, 18:0/18:1 PA, and di-18:1 PA) activate mTOR complex 1 (mTORC1) in mitogenic stimulation, suggesting the unique role of PLD1-produced PA species in mTORC1 regulation^13^. In addition, the involvement of PLD1 in liver disease and insulin signaling was previously reported; nuclear ARF dependent PLD activity increases during S-Phase of rat liver regeneration^14^, and PLD1 plays a role in the development and progression of rat liver fibrosis^15^,^16^. Additionally, PLD1 is activated by insulin in rat hepatocytes^17^ and regulates insulin-stimulated fusion of localized glucose transporter type 4 (GLUT4)-containing vesicles to the plasma membrane, resulting in an increase in glucose uptake by the adipocyte^18^. Nevertheless, the precise roles of PLD1 and its product, PA in NAFLD and insulin resistance have not been examined.

Herein, we used *Pld1*^−/−^ mice to investigate the role of PLD1 in NAFLD and its consequent insulin resistance. Our results strongly suggested that PLD1 deficiency impairs autophagy, resulting in the accumulation of lipids in the liver, without affecting insulin resistance.

## Results

### PLD1 is downregulated in NAFLD

**Figure 1:**
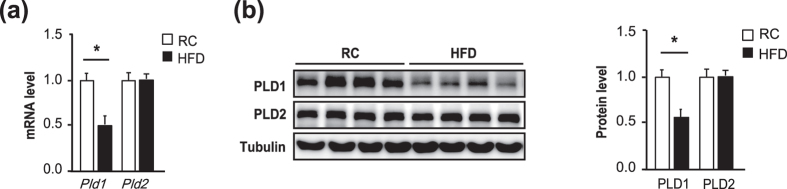
*Pld1* expression is decreased in HFD-induced hepatic steatosis. (**a**) C57BL/6 mice were fed either RC or a HFD for 4 weeks (n = 10 per group) starting at the age of 13 weeks and fasted overnight prior to collection of the liver. mRNA was isolated from the liver for qRT-PCR. (**b**) Mice were maintained as in (**a**) and the lysates were analyzed by western blot. Western blot results were analyzed by densitometry to obtain the relative ratio of either PLD1 or PLD2 to tubulin. The data are presented as means ± SE or representative blots from 3 to 5 independent experiments. *P < 0.05 versus the RC group. Abbreviations: HFD, high-fat diet; PLD2, phospholipase D2; RC, regular chow; SE, standard error; qRT-PCR quantitative real-time polymerase chain reaction.

To examine the involvement of PLD in NAFLD, we compared the expression levels of PLD between the liver and other metabolism-related organs, such as skeletal muscle, epididymal fat, and brown adipose tissue (BAT). PLD1 and PLD2 were highly expressed in the liver, but not in other organs (Supplementary Fig. 1). Next, we compared *Pld* expression levels between the liver of high-fat diet (HFD)-fed mice with hepatic steatosis and mice fed regular chow (RC) without hepatic steatosis to validate the relevance of PLD in NAFLD. The *Pld1* transcript (Fig. 1a) and protein (Fig. 1b) levels were significantly lower in mice with HFD-induced hepatic steatosis than in mice fed RC, whereas *Pld2* expression did not differ between the groups. Thus, PLD1, but not PLD2, could be related to hepatic steatosis.

### PLD1 deficiency induces NAFLD

**Figure 2:**
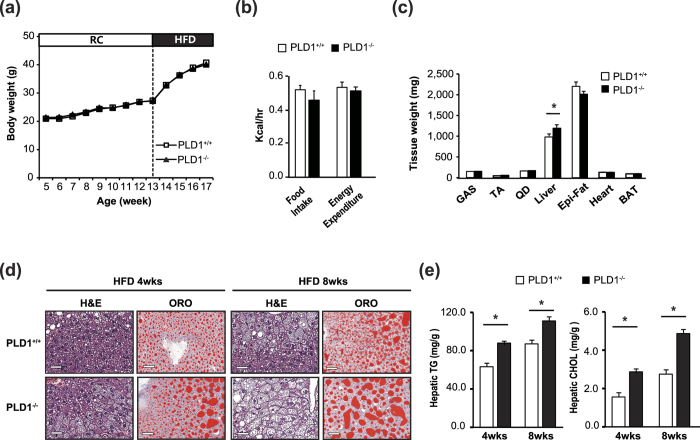
*Pld1*^−/−^ mice have hepatic steatosis. (**a**) *Pld1*^+/+^ and *Pld1*^−/−^ littermates were fed HFD for 4 weeks starting at the age of 13 weeks (n = 9–11 per group). Body weights were measured. (**b**) The mice were fed HFD for 2 weeks starting at the age of 13 weeks. The food intake and energy expenditure of the mice were measured using indirect calorimetry over a 24-h period. (**c–e**) *Pld1*^+/+^ and *Pld1*^−/−^ littermates were fed HFD for either 4 or 8 weeks starting at the age of 13 weeks (n = 9–11 per group). (**c**) The weights of each tissue were measured. (**d**) The livers were isolated and frozen sections were stained with H&E (left) and ORO (right). Representative images were obtained at 200× magnification. Scale bar, 50 μm. (**e**) Hepatic triglycerides and cholesterol were quantitatively analyzed. The data are presented as means ± SE or representative images from 3 to 5 independent experiments. *P < 0.05 versus the *Pld1*^+/+^ mice. Abbreviations: BAT, brown adipose tissue; GAS, gastrocnemius; HFD, high-fat diet; H&E, hematoxylin and eosin; ORO, Oil Red O; SE, standard error; TA, tibialis anterior; QD, quadriceps.

We used *Pld1*^−/−^ mice to examine the role of PLD1 in hepatic steatosis. We used *Pld1*^+/+^ littermates as control throughout the study. The elimination of PLD1 protein expression in liver tissues was confirmed (Supplementary Fig. 2A), as shown previously^19^. *Pld1*^−/−^ mice were fed HFD for 4 weeks starting at 13 weeks of age. The body weight of *Pld1*^−/−^ mice did not differ from that of their *Pld1*^+/+^ littermates fed both, RC and a HFD (Fig. 2a). There were no differences in food intake and energy expenditure, as measured by CLAMS (Fig. 2b). Additionally, there was no difference in body composition (body weight, fat, and muscle) between *Pld1*^+/+^ mice and *Pld1*^−/−^ mice (Supplementary Fig. 2B). To further analyze body composition, we measured the weight of individual tissues. As shown in Fig. 2c, the liver was significantly heavier in *Pld1*^−/−^ mice than *Pld1*^*++*^ mice, whereas the weights of other tissues did not differ between mice. A histological analysis using hematoxylin and eosin (H&E) and Oil Red O staining showed enhanced intracellular vacuolation and lipid accumulation in both 4- and 8-week HFD-fed *Pld1*^−/−^ mice (Fig. 2d). Consistent with these observations, hepatic TG and cholesterol increased by 26.5% and 60.4%, respectively, in 4-week HFD-fed *Pld1*^−/−^ mice as well as by 22.4% and 75%, respectively, in 8-week HFD-fed *Pld1*^−/−^ mice (Fig. 2e). In addition, there was no difference in the size of adipocytes in the epididymal fat (Supplementary Fig. 2C), suggesting that lipid accumulation was specific to the liver. Moreover, alanine transaminase (ALT) and aspartate transaminase (AST) activity, which are two of the most reliable markers of hepatocellular injury^20^, were 45.6% and 23.4% higher, respectively, in the serum of *Pld1*^−/−^ mice than in *Pld1*^+/+^ mice, indicating that liver dysfunction occurred in the *Pld1*^−/−^ mice (Table 1). These data indicate that PLD1 deficiency specifically induces lipid accumulation in the liver.

### Fat oxidation is decreased in the liver of *Pld1* ^−/−^ mice

**Figure 3:**
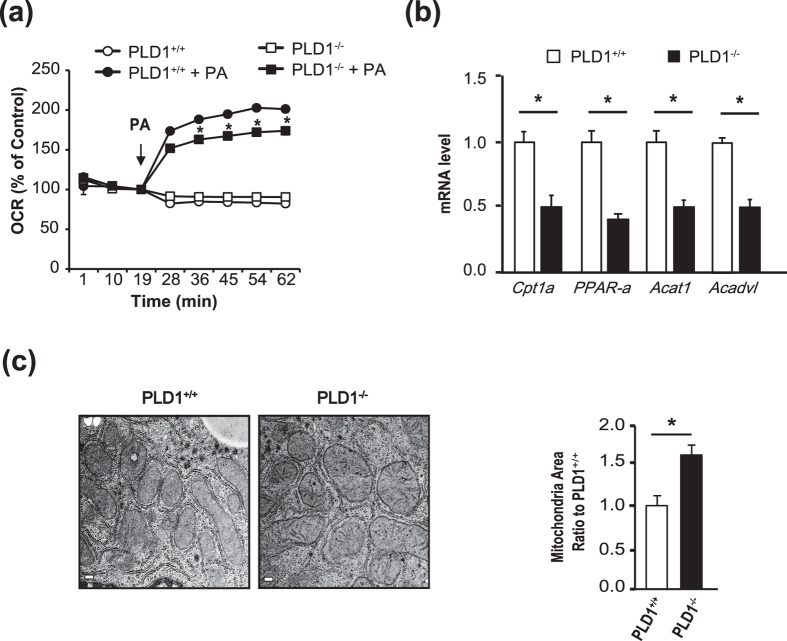
The fat oxidation rate is reduced in the livers of *Pld1*^−/−^ mice. (**a**) Primary hepatocytes were isolated from *Pld1*^+/+^ and *Pld1*^−/−^ littermates, serum starved, and treated with bovine serum albumin/palmitate complexes for 2 h. The oxygen consumption rate was measured at the indicated time points (n = 5 per each group). (**b**) The *Pld1*^+/+^ and *Pld1*^−/−^ littermates were fed a HFD for 4 weeks starting at the age of 13 weeks (n = 9–11 per group) and qRT-PCR was performed. (**c**) The mice were maintained as described in (**b**) and the livers were collected. The mitochondria in the livers from *Pld1*^+/+^ and *Pld1*^−/−^ littermates were visualized by transmission electron microscopy. The mitochondrial area was quantitatively measured using ImageJ (n = 3 mice per group). At least 15 pictures per group were evaluated. Scale bar, 0.2 μm. The data are presented as means ± SE or representative images from 3 to 5 independent experiments. *P < 0.05 versus the *Pld1*^+/+^ group. Abbreviations: HFD, high-fat diet; PA, palmitic acid; qRT-PCR, quantitative real-time polymerase chain reaction; SE, standard error.

Hepatic lipid content is controlled by a balance between hepatic lipid uptake, lipid synthesis, fat oxidation (or lipolysis), and lipid export^21^, suggesting that NAFLD is caused by an imbalance in lipid supply and demand. To investigate the mechanism by which PLD1 deficiency induces NAFLD, we tested whether the *Pld1*^−/−^ mice had a lipid flux defect. We did not observe differences in the expression of the relevant genes between *Pld1*^−/−^ and *Pld1*^+/+^ mice (Supplementary Fig. 3A). The TG levels in *Pld1*^−/−^ mice after the injection of the lipoprotein lipase inhibitor poloxamer during fasting were comparable to those in their *Pld1*^+/+^ littermates (Supplementary Fig. 3B), indicating that VLDL secretion was normal in *Pld1*^−/−^ mice. Furthermore, the levels of genes involved in lipid synthesis and PA metabolism were not significantly altered in the *Pld1*^−/−^ mice (Supplementary Fig. 3C). Next, we examined the oxygen consumption rate (OCR) in primary hepatocytes to assess mitochondrial function. As shown in Fig. 3a, the OCR did not differ between *Pld1*^+/+^ and *Pld1*^−/−^ hepatocytes in the basal state; however, it was significantly lower in *Pld1*^−/−^ hepatocytes than in *Pld1*^+/+^ hepatocytes after palmitic acid loading. Furthermore, the expression levels of fatty acid oxidation-related genes were significantly decreased in *Pld1*-null primary hepatocytes (Fig. 3b). *Pld1*^−/−^ livers had swollen mitochondria under fasting conditions and a significant increase was observed in the mitochondrial area in *Pld1*^−/−^ compared with *Pld1*^+/+^ mice (Fig. 3c). Together, these results strongly suggest that lipid accumulation in the *Pld1*-null liver results from decreased fat oxidation and impaired mitochondrial function.

### *Pld1* ^−/−^ mice have an autophagy defect

**Figure 4:**
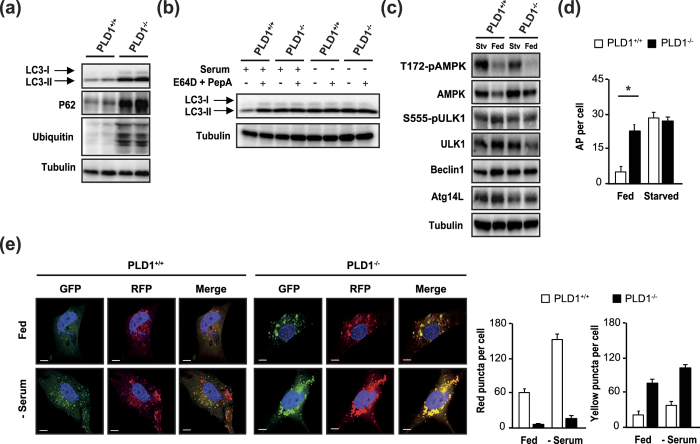
Autophagy is impaired in the livers of *Pld1*^−/−^ mice. (**a**) Primary hepatocytes were isolated from *Pld1*^+/+^ and *Pld1*^−/−^ littermates, cultured in a serum-fed state, and the cell lysates were analyzed by western blotting. (**b**) Primary hepatocytes isolated as described in (**a**) were treated with or without E64D (10 μg/ml) and PepA (10 μg/ml) under either serum-fed or serum-starved conditions for 24 h. The cell lysates were analyzed by western blot. (**c**) Livers were collected from *Pld1*^+/+^ and *Pld1*^−/−^ littermates were fed HFD for 4 weeks (n = 9–11 per group) under either fed or starved conditions for 24 h. Liver lysates were analyzed by western blot. (**d**) Livers were collected as described in (**c**). APs were quantitatively analyzed in transmission electron microscopic images of the liver. (**e**) *Pld1*^+/+^ and *Pld1*^−/−^ MEF were infected with RFP-GFP-LC3B (60 particles per cell) for 24 h and then assigned to either serum-fed or serum-starved conditions. Representative confocal microscopy images are shown. GFP-negative/RFP-positive and GFP-positive/RFP-positive puncta were counted using ZEN software; more than 80–90 cells were included for each of 3 independent experiments. Scale bar, 20 μm. The data are presented as means ± SE or representative blots from 3 to 5 independent experiments. *P < 0.05 versus *Pld1*^+/+^ mice. Abbreviations: GFP, green fluorescence protein; HFD; MEF, mouse embryonic fibroblasts; PepA, pepstatin A; RFP, red fluorescence protein; SE, standard error.

Autophagy regulates hepatic lipid stores *in vivo*, and defects in lipolysis are accompanied by defects in autophagy^6^. Autophagy-related gene (ATG)-deleted mouse models exhibit mitochondrial swelling and reduced respiration in the liver^22^, similar to our observations in *Pld1*^−/−^ liver. In addition, PLD1 is involved in macroautophagy^19^. Therefore, we examined autophagy flux in *Pld1*^−/−^ mice to determine the mechanism underlying NAFLD. Surprisingly, microtubule-associated protein 1 light chain 3 alpha (LC3)-I/II, P62, and polyubiquitinated protein expression increased in hepatocytes isolated from *Pld1*^−/−^ mice (Fig. 4a), indicating a decrease in autophagic protein degradation. To validate the defect in autophagic degradation in *Pld1*^−/−^ hepatocytes, we treated cells with the lysosomal protease inhibitors E64D and pepstatin A (PepA). The LC3-I/II levels in *Pld1*^+/+^ hepatocytes were increased by E64D and PepA treatment in the serum-fed state to levels similar to those observed for serum starvation (Fig. 4b). In contrast, the elevated LC3-I/II levels in *Pld1*^−/−^ hepatocytes in the serum-fed state were not increased upon serum starvation or by E64D and PepA treatment, indicating that autophagic flux was impaired in *Pld1*^−/−^ mice (Fig. 4b). However, PLD1 deficiency did not alter autophagy signaling, as shown by the expression level of autophagic regulators such as ATG14L and Beclin1, and the phosphorylation of ULK1 at serine 555 and AMPK at threonine 172 (Fig. 4c). These results suggest that the PLD1 deficiency did not regulate autophagy by regulating the autophagic signaling. In addition, an electron microscopy analysis of the liver showed that autolysosomes (dense structures with membranes) were reduced in *Pld1*^−/−^ mice and autophagosomes (AP) with lipids (light structures with membranes) were primarily observed in *Pld1*^−/−^ mice (Supplementary Fig. 4). In agreement with these results, the number of APs was higher in liver of fed *Pld1*^−/−^ mice (Fig. 4d), indicating that PLD1 deficiency decreased the fusion of APs to lysosomes, resulting in AP accumulation. To confirm the defect in autolysosome formation in *Pld1*^−/−^ mouse embryonic fibroblasts (MEFs), we attempted to differentiate between APs and autolysosomes using an RFP-GFP-LC3 construct. Because RFP is more stable than GFP under acidic conditions, only RFP maintains its fluorescence upon AP and lysosome fusion, resulting in an increase in red puncta. Red puncta were observed in *Pld1*^+/+^ MEFs in the serum-fed state, establishing a basal autolysosome level (Fig. 4e). Serum starvation increased the numbers of both red and yellow (merge of green and red) puncta, indicating increases in both APs and autolysosomes. However, the *Pld1*^−/−^ MEFs contained only a few red puncta and many large-sized yellow aggregates in both serum-fed and -starved states (Fig. 4e). These results demonstrate that *Pld1*^−/−^ MEFs had a defect in the fusion of autolysosomes, rather than in the formation of APs.

### The PLD1 product PA regulates autophagy

**Figure 5:**
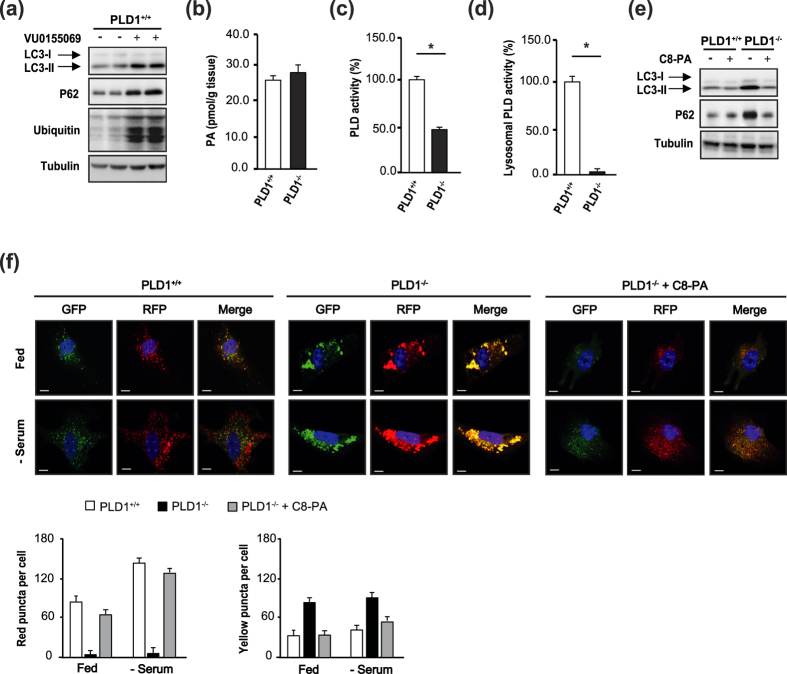
PA restores autophagy. (**a**) Primary hepatocytes were isolated from *Pld1*^+/+^ and *Pld1*^−/−^ littermates, treated with 5 μM VU0155069 in serum-fed conditions for 24 h, and the lysates were analyzed by western blot. (**b**) The *Pld1*^+/+^ and *Pld1*^−/−^ littermates were fed HFD for 4 weeks starting at the age of 13 weeks (n = 3 per group), livers were collected, and total phosphatidic acid levels were measured by LC-MS/MS. (**c,d**) The mice were fed as described in (**b**). (**c**) The lysates and (**d**) the lysosomal fractions of livers were isolated and a PLD assay was performed using a PLD assay kit. (**e**) Primary hepatocytes were isolated from *Pld1*^+/+^ and *Pld1*^−/−^ littermates and treated with or without 300 μM C8-PA in serum-fed conditions for 24 h; the lysates were analyzed by western blot. (**f**) *Pld1*^+/+^ and *Pld1*^−/−^ MEFs were infected with RFP-GFP-LC3B (60 particles per cell) for 24 h. *Pld1*^+/+^ MEFs, *Pld1*^−/−^ MEFs, and *Pld1*^−/−^ MEFs treated with 300 μM C8-PA were maintained in either serum-fed or serum-starved conditions for 24 h. Representative confocal microscopy images from 3–5 independent experiments are shown. The average numbers of GFP-negative/RFP-positive or GFP- and RFP-positive cells were counted using ZEN software from three independent experiments (60 cells per experiment). The data are presented as means ± SE or representative blots from 3 to 5 independent experiments. *P < 0.05 versus *Pld1*^+/+^ mice. Abbreviations: C8-PA, C8-phosphatidic acid; GFP, green fluorescence protein; HFD, high-fat diet; LC-MS/MS, liquid chromatography–mass spectrometry/mass spectrometry; MEF, mouse embryonic fibroblasts; RFP, red fluorescence protein; SE, standard error.

To determine whether PLD1 activity is required to regulate autophagy in the liver, we treated primary hepatocytes with the PLD1-specific inhibitor VU0155069. As shown in Fig. 5a, LC3-I/II, P62, and polyubiquitinated proteins were elevated in VU0155069-treated *Pld1*^+/+^ hepatocytes, consistent with the observations in *Pld1*^−/−^ hepatocytes. Next, we tested whether PLD1 deficiency indeed decreased PA levels in cells. Unexpectedly, neither PA (Fig. 5b, Supplementary Fig. 5A) nor other anionic phospholipid levels (Supplementary Fig. 5B) were altered in *Pld1*^−/−^ liver as compared to controls. We measured PLD activity in the liver and lysosomal fractions of the liver (the primary site of PLD1 localization^23^ and autophagolysosome formation). PLD activity in *Pld1*^−/−^ liver was not completely eliminated owing to PLD2 activity (Fig. 5c). Notably, PLD activity was almost completely dampened in the lysosomal fraction of the *Pld1*^−/−^ liver (Fig. 5d), suggesting that PA in lysosomes is predominantly produced by PLD1, and not by PLD2, and PLD1-produced PA may play a critical role in the regulation of autophagy. To confirm that PA is required for autophagic flux, we treated *Pld1*^−/−^ hepatocytes in the serum-fed state with C8-PA. C8-PA treatment restored the autophagic flux in *Pld1*^−/−^ hepatocytes, as evidenced by decreases in LC3-I/II and P62 to levels similar to those of *Pld1*^+/+^ hepatocytes (Fig. 5e). Consistent with this observation, C8-PA treatment increased the number of red puncta in the *Pld1*^−/−^ MEFs and reduced the number of large yellow aggregates (Fig. 5f), confirming that PA is critical for autophagy.

### Hepatic PLD1 expression in *Pld1* ^−/−^ mice attenuates hepatic steatosis

**Figure 6:**
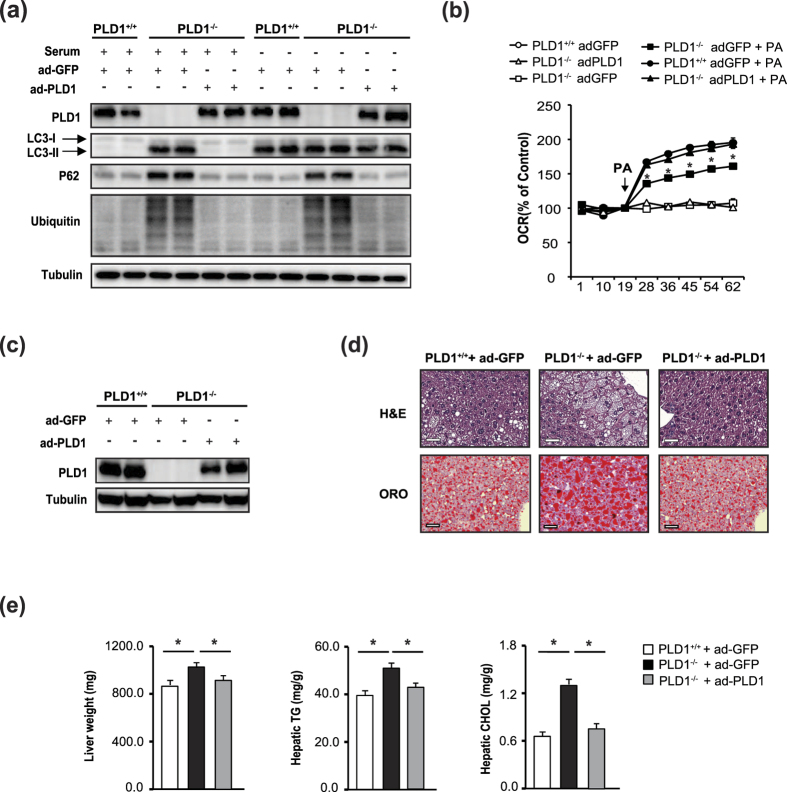
Hepatic PLD1 expression in *Pld1*^−/−^ mice attenuates hepatic steatosis. (**a**) Primary hepatocytes were isolated from *Pld1*^+/+^ and *Pld1*^−/−^ littermates, infected with adGFP or adPLD1 for 24 h, and treated with or without serum for 24 h. Western blot was performed with cell lysates. (**b**) Primary hepatocytes were infected with adGFP or adPld1 for 24 h, serum starved, and then treated with bovine serum albumin/palmitate complexes for 2 h. The OCR was measured at the indicated time points. (**c**) After feeding the mice with a HFD for one week beginning at 13 weeks, the mice were injected with adGFP or adPld1 (1 × 10^9^ PFU) by tail vein injection. At 12 days post-injection, mice were sacrificed for analyses. Mouse liver lysates were analyzed by western blotting. (**d**) Mice were maintained as described in (**c**) and the livers were isolated, and frozen sections were stained with H&E (left) and ORO (right). Representative images were obtained at 200× magnification from 3–5 independent experiments. Scale bar, 50 μm. (**e**) Mice were maintained as described in (**c**); the weights of livers from mice were measured and hepatic triglycerides and cholesterol were quantitatively analyzed. The data are presented as means ± SE or representative blots from 3 to 5 independent experiments. *P < 0.05 versus *Pld1*^+/+^ mice. Abbreviations: adGFP, adenovirus-expressing green fluorescence protein; adPld1, adenovirus-expressing phospholipase D1; H&E, hematoxylin and eosin; HFD,; OCR, oxygen consumption rate; PA, palmitic acid; PFU, plaque-forming units; SE, standard error.

To clarify the role of PLD1 in autophagy and hepatic steatosis, we expressed PLD1 using adenoviral gene transfer. We used adenoviral GFP as a control to remove non-specific effects of adenoviral infection. Consistent with previous reports in which autophagic flux decreased^24^,^25^, LC3-I/II, P62, and ubiquitinated proteins in *Pld1*^−/−^ hepatocytes were elevated in both serum-fed and serum-starved conditions, indicating the blockage of autophagic flux (Fig. 6a). In serum-starved conditions, autophagic flux in *Pld*^+/+^ hepatocytes increased; LC3-I/II increased, and there were no changes in the levels of P62 and ubiquitinated proteins compared to serum-fed conditions (Fig. 6a). However, PLD1 expression restored autophagic flux to a level similar to that in *Pld1*^+/+^ hepatocytes in both serum-fed and -starved conditions (Fig. 6a). PLD1 expression in *Pld1*^−/−^ hepatocytes dampened the elevations in LC3-I/II, P62, and ubiquitinated protein expression in serum-fed conditions, and P62 and ubiquitinated proteins in serum-starved conditions, suggesting that PLD1 expression in *Pld1*^−/−^ hepatocytes restored autophagic flux (Fig. 6a). Consistent with the augmentation of autophagy in PLD1-expressing *Pld1*^−/−^ hepatocytes, the OCR was recovered in these cells (Fig. 6b), indicating that the defect in fat oxidation might be related to the autophagic defect in *Pld1*^−/−^ hepatocytes. When PLD1 expression was restored in *Pld1*^−/−^ livers using adenoviral gene transfer via tail vein injection (Fig. 6c), hepatic lipid accumulation was dramatically reduced (Fig. 6d), accompanied by significant decreases in liver weight, hepatic TGs, and hepatic cholesterol (Fig. 6e). Despite the successful PLD1 expression by adPLD1 in *Pld1*^−/−^ livers, adPLD1 did not further induce PLD1 expression in *Pld1*^+/+^ livers, and was not accompanied by any changes in liver weight, hepatic TG, or cholesterol (Supplementary Fig. 6A,B). These results indeed confirm that PLD1 is a critical regulator of hepatic lipid accumulation by controlling autophagy.

### *Pld1* ^−/−^ mice exhibit normal insulin sensitivity

**Figure 7:**
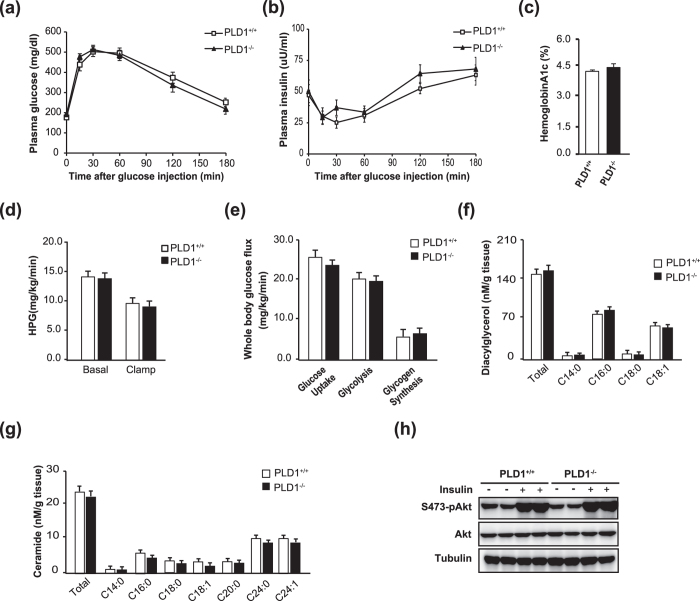
Hepatic steatosis in *Pld1*^−/−^ mice is dissociated from insulin resistance. The mice were fed HFD for 4 weeks at 13 weeks. (**a,b**) The mice were fasted overnight and injected with glucose (IP, 1 g/kg body weight). (**a**) The plasma glucose level was analyzed at the indicated time points. (**b**) Plasma insulin was measured during IPGTT. (**c**) Hemoglobin A1c was measured in whole blood from mice. (**d**) Hepatic glucose production and (**e**) the whole body glucose flux were measured before and during clamp. (**f**) The ceramide and (**g**) diacylglycerol levels in the livers of *Pld1*^+/+^ and *Pld1*^−/−^ mice were measured by LC/MS. (**h**) The mice were fasted overnight, injected with either saline or insulin (IP, 1.5 units/kg; n = 5 per group), and sacrificed 10 min after injection. Liver lysates were analyzed by western blot. The results are shown as means ± SE or representative blots from 3 to 5 independent experiments. *P < 0.05 versus *Pld1*^+/+^ mice. Abbreviations: IPGTT, intraperitoneal glucose tolerance test; LC/MS, liquid chromatography–mass spectrometry; SE, standard error.

Hepatic steatosis is associated with insulin resistance, which is a major factor in the pathogenesis of type 2 diabetes^26^. To explore the role of hepatic steatosis in insulin resistance in *Pld1*^−/−^ mice, we analyzed the glucose response using an intraperitoneal glucose tolerance test (IPGTT). Glucose clearance was similar in *Pld1*^+/+^ and *Pld1*^−/−^ mice (Fig. 7a). In addition, there were no significant differences in insulin levels during IPGTT (Fig. 7b) or hemoglobin A1c (Fig. 7c). To examine the differences in whole body and tissue-specific insulin sensitivities between *Pld1*^+/+^ and *Pld1*^−/−^ mice, a hyperinsulinemic-euglycemic clamp study was conducted. The steady-state blood glucose level (Supplementary Fig. 7A), glucose infusion rate (Supplementary Fig. 7B), hepatic glucose production rate (Fig. 7d), and the rates of whole body glucose uptake, glycolysis, and glycogen synthesis (Fig. 7e) in *Pld1*^−/−^ mice were not significantly different from those in *Pld1*^+/+^ mice. To better understand normal insulin sensitivity despite hepatic steatosis in *Pld1*^−/−^ mice, we analyzed lipid metabolite levels in the livers of *Pld1*^+/+^ and *Pld1*^−/−^ mice because elevated ceramide and DAG levels are associated with insulin resistance^27^,^28^,^29^. As shown in Fig. 7f,g, neither total DAG nor ceramide showed differences between the *Pld1*^−/−^ and *Pld1*^+/+^ mice. Finally, insulin administration similarly increased Akt phosphorylation in the liver (Fig. 7h), skeletal muscle, and white adipose tissue (Supplementary Fig. 7C), confirming the comparable insulin sensitivity in *Pld1*^−/−^ and *Pld1*^+/+^ mice. Taken together, our results demonstrate that hepatic steatosis in *Pld1*^−/−^ mice is not coupled with insulin resistance.

## Discussion

Although PLD has known roles in cancer and inflammation^30^, the relevance of PLD1 in NAFLD remains unclear. We provide the first evidence that PLD1 is a critical determinant of NAFLD. Specifically, the loss of PLD1 impaired autophagic flux via defects in the fusion between APs and lysosomes, resulting in NAFLD. Furthermore, we showed that PLD1 deficiency-induced NAFLD was not associated with obesity or insulin sensitivity.

Autophagy involves the delivery of intracellular cargo to lysosomes for degradation^31^. Basal autophagy performs a housekeeping function by removing damaged components. During nutrient starvation, autophagy supplies nutrients for survival by degrading proteins, lipids, and organelles. Hepatic autophagy breaks down lipid droplets to provide free fatty acids for oxidation to produce ATP in response to an increase in lipid availability or nutrient deprivation^6^. Defective autophagy in mice results in larger lipid droplets and increases hepatic triglyceride levels and gross liver size^31^, in agreement with our observations in *Pld1*^−/−^ mice (Fig. 2). Fat accumulation in *Pld1*^−/−^ liver was comparable to that in *Vps34*^−/−^ 25 and *Atg5*^−/−^ livers^22^. Indeed, autophagic flux and the fat oxidation (β-oxidation) rate were significantly reduced, whereas de novo fat synthesis, fat uptake, and VLDL secretion were unchanged in *Pld1*^−/−^ mice. Decreased β-oxidation rate and swollen mitochondria, which were observed in *Pld1*^−/−^ livers, are correlated with defective autophagy is other mouse models^25^, suggesting that defective autophagy in the *Pld1*^−/−^ mice caused the mitochondrial abnormalities and subsequent lipid accumulation.

Previous studies have shown that PLD1 is a positive regulator of autophagy^19^,^32^, even though an independent study suggested that PLD1 negatively regulates autophagy via the coordination of important molecules in the autophagic pathway, AMPK-mTOR-ULK1 and Vps34/Beclin 1^33^. Dall’Armi *et al*. proposed that PLD1 functions as a regulator of AP dynamics during starvation-induced autophagy^19^. They observed that a significant pool of PLD1 localizes on LAMP1-positive late endosomes/lysosomes in normal medium, consistent with previous studies^23^,^34^. They found that under nutrient starved conditions, PLD1 is transferred from late endosomes/lysosomes to APs, and functions during maturation as an effector of Vps34, a class III PI3 kinase. Unlike their study, in which PLD1 localization on APs is important for AP maturation during autophagy, we found that PLD1 activity on lysosomes is critical for the regulation of autophagy in the liver. We observed that the decrease in lysosomal PLD activity in *Pld1*^−/−^ livers dampened the fusion between APs and lysosomes, suggesting that PLD1-generated PA on lysosomes is required for AP-lysosome fusion (Figs 4 and 5). Indeed, PA treatment in *Pld1*-null hepatocytes increased fusion between the AP and the lysosome, as shown by the increase in the number of autophagolysosomes and decreases in LC3-II, P62, and ubiquitinated protein levels (Fig. 5). Nevertheless, it is possible that PA on APs is required for AP-lysosome fusion together with PA on lysosome, which is worthy of further investigation.

Recently, autophagy activation in POMC neurons was reported to elicit lipid utilization in peripheral tissues such as BAT and liver tissue, by activating both lipophagy and cytosolic lipases, suggesting that autophagy in the central nervous system regulates lipid accumulation in peripheral tissues^35^. They showed that autophagy signaling from the brain increases the expression of ATGs in BAT and liver tissues, and AMPK-induced ULK1 phosphorylation in BAT. PLD1 inhibition in the brain decreases autophagic flux^32^; therefore, it is possible that PLD1 depletion in the brain prompts lipid accumulation in *Pld1*^−/−^ liver. However, we observed a defect in the fusion process between APs and lysosomes, without changes in the expression of Atg14L or Beclin1, or in the phosphorylation of AMPK and ULK1 (Fig. 4c), implying that lipid accumulation in *Pld1*^−/−^ livers is not caused by autophagic defects from the central nervous system.

Based on the decrease in lysosomal PA in *Pld1*^−/−^ mice, transient cellular PA kinetics^36^, and the absence of other lipid-converting enzymes in lysosomes^37^,^38^, the conversion of PC to PA by PLD1 may be the main determinant of lysosomal PA levels, rather than the conversion of other lipids such as DAG. Indeed, other PA-generating enzymes, such as GPAT/AGPAT and DGK, are localized on the endoplasmic reticulum and mitochondrial membranes^37^ and are unlikely to regulate the lysosomal PA level. In contrast to the lack of differences in PA levels observed in our deficiency model, the overexpression of *Pld1* in a whole cell system increases the total PA level^39^. Elevated PA levels in *Pld1*-overexpressing hepatocytes inhibit Akt by disrupting mTORC2^39^, which localizes to the plasma membrane^40^. Based on the different localization patterns of mTORC2 and PLD1, the lysosomal deficiency of PA probably does not regulate insulin-stimulated Akt via mTORC2.

NAFLD is associated with hepatic insulin resistance, which is a major risk factor for type 2 diabetes^36^. One potential mechanism underlying fat-induced hepatic insulin resistance is the accumulation of various lipid metabolites (especially DAG and ceramide) in the liver. This accumulation may activate several protein kinase cascades such as protein kinase C (PKC)-ε, resulting in the disruption of insulin signaling^41^,^42^,^43^,^44^. In our study, total content of DAG and ceramide, which are the most probable candidate lipid metabolites associated with insulin resistance, did not differ between *Pld1*^−/−^ and *Pld1*^+/+^ liver (Fig. 7f,g). As expected, Akt phosphorylation in *Pld1*^−/−^ liver did not differ from that in *Pld1*^+/+^ liver (Fig. 7h). These results provide another example of the dissociation between hepatic steatosis and insulin resistance and explain the underlying mechanisms^45^,^46^,^47^,^48^,^49^. Recently, Trujillo Viera *et al*. reported that *Pld1*^−/−^ mice consume more food due to defects in the hypothalamus, which results in obesity^50^. They observed obvious obesity in 20-week old *Pld1*^−/−^ mice, followed by increase in free fatty acid (FFA) levels and glucose levels in the blood of *Pld1*^−/−^ mice. Hence, they proposed that elevation of FFA decreased insulin sensitivity in *Pld1*^−/−^ mice. However, in our current study, we did not observe any changes in FFA (Table 1) and glucose levels (Supplementary Fig. 7A) in the blood of *Pld1*^−/−^ mice or in insulin sensitivity in *Pld1*^−/−^ liver (Fig. 7d,e,h) of *Pld1*^−/−^ mice fed HFD for 4 weeks starting at 13 weeks of age. Since Trujillo Viera *et al*. proposed that FFA elevation due to obesity induced insulin resistance, we believe that lack of obesity in *Pld1*^−/−^ mice in our study was responsible for the difference in our findings. The precise reasons for the discrepancies between our data and those of Trujillo Viera *et al*. are difficult to be certain of; all we can say is that they studied the mice at different age from ours and on a different diet formulation (Regular Chow versus High Fat Diet) and laboratory environment.

In summary, the results of this study demonstrate that a decrease in PA production by lysosomal PLD1 inhibits autolysosome formation in the *Pld1*^−/−^ liver, leading to hepatic steatosis, without affecting insulin sensitivity. Thus, deciphering the specific function of lysosomal PLD1 is essential for the identification of new therapeutic strategies for hepatic steatosis.

## Materials And Methods

### Animals

*Pld1*^−/−^ mice were generated and characterized as previously described^19^. Littermates that were homozygous for the PLD1 allele were used as controls. Mice were housed under controlled temperature (22 °C ± 2 °C) and 12-h light-dark cycle, and fed HFD (60 kcal% fat diet for 4 and 8 weeks; D12492 obtained from Research Diet Incorporation). All experimental protocols for animals, maintenance and care, were conducted according to Gachon University Animal Care guidelines. All animal procedures were approved by the Center of Animal Care and Use, Lee Gil Ya Cancer and Diabetes Institute, Gachon University and the Institutional Animal Care and Use Committee (IACUC) (Permission number: LCDI-2016-0008).

### Basal study

Fat and lean body mass were assessed using an ^1^H minispec system (Bruker BioSpin) before and after 4 weeks on HFD. Activity, food consumption, and energy expenditure were evaluated using a metabolic monitoring system (CLAMS; Columbus Instruments, Columbus) for 4 days (2 days of acclimation followed by 2 days of measurement) before and after 2 weeks on HFD, as previously described by Choi *et al*.^51^. For the glucose tolerance test and lipoprotein inhibitor assay, mice were fasted overnight before either intraperitoneal injection of glucose (1.5 g/kg body weight) or Poloxamer 407 (1 g/kg body weight). Blood glucose and insulin were analyzed from tail-vein blood collected at the indicated time points. To examine the insulin signaling pathway, insulin (1.0 U/kg body weight) was injected into the peritoneal cavity. Liver, muscle, and epididymal fat were isolated 10 min after the injection. For the analyses of gene expression and lipid metabolites, mice were fasted for 16 h and the blood and tissue samples were immediately collected.

### Hyperinsulinemic-euglycemic clamp

To assess whole body and hepatic insulin sensitivity, we performed a hyperinsulinemic-euglycemic clamp study as previously described by Choi *et al*.^52^. Indwelling catheters were placed in the internal jugular vein 7 days before the hyperinsulinemic-euglycemic clamp studies. After overnight fasting, [3-^3^H] glucose (Perkin Elmer) was infused at a rate of 0.05 μCi/min for 2 h to measure the basal glucose turnover. After 2 h, a hyperinsulinemic-euglycemic clamp was used for 150 min with an infusion of insulin (3 mU/kg/min). Blood samples (10 μl) were collected at the indicated time points to analyze plasma glucose, and 20% dextrose was infused at various rates to keep plasma glucose at basal levels (120 mg/dl). To assess whole body glucose fluxes, [3-^3^H] glucose was infused at a rate of 0.1 μCi/min throughout the clamps. To estimate insulin-stimulated glucose uptake and metabolism in peripheral tissues, 2-deoxy-d-[1-^14^C] glucose (Perkin Elmer) was administered as a bolus at 125 min throughout the clamps. To determine ^3^H and ^14^C activity levels in the plasma, blood samples were taken at the basal period and during the last 60 min of the clamp.

### Isolation of lysosomal fractions and PLD activity assay

Lysosomal fractions were isolated from the liver using a Lysosome Isolation Kit (LYSISO1, Sigma Aldrich) according to the manufacturer instructions. PLD activity was measured using Phospholipase D Assay Kit (MAK137, Sigma Aldrich) according to the manufacturer instructions.

### Preparation and culture of primary hepatocytes and mouse embryonic fibroblasts (MEFs)

To prepare MEFs, 13–15-d post-coital mouse embryos were minced and digested with trypsin. The cells were collected and cultured in Dulbecco’s modified Eagle’s medium with 4.5 mg/l glucose, 10% fetal bovine serum [FBS], 100 U/ml penicillin, and 100 μg/ml streptomycin. To prepare primary hepatocytes, 8 to 10-week-old male mice were anesthetized and their livers were isolated. Primary hepatocytes were prepared by 2-step perfusion using liver perfusion medium and liver digestion buffer (Life Technologies, 17701038, 17703034) as previously described^53^. Primary hepatocytes were cultured on tissue culture dishes in M199 medium (Sigma Aldrich, M4530) containing 10% FBS, 100 U/ml penicillin and streptomycin, and 10 nM dexamethasone.

### Measurement of oxygen consumption rate

The rate of oxygen consumption (OCR) was measured using an extracellular Flux Analyzer XF-24 (Seahorse Biosciences). Primary mouse hepatocytes were seeded into 24-well plates (2 × 10^4^/plate) and cultured overnight in M199 media with 10% FBS. Cells washed with Krebs-Henseleit buffer (111 mM NaCl, 4.7 mM KCl, 2 mM MgSO_4_, 1.2 mM Na_2_HPO_4_, 2.5 mM glucose, and 0.5 mM carnitine) and incubated in the same buffer for 1 h at 37 °C. Cells were then loaded onto the XF24 analyzer to measure the OCR in short and repeated intervals. After injection with BSA (17 μM) or BSA-complexed palmitic acid (final concentration 150 μM), mixing, waiting and OCR were measured at indicated time points.

### Western blot analyses

Cells and tissue proteins were lysed in cell lysis buffer (Cell Signaling Technology, 9803) containing phosphatase inhibitors and protease inhibitors (Sigma Aldrich, P5726, P2714). Twenty micrograms of protein were used for western blot. The proteins were resolved on sodium dodecyl sulfate-polyacrylamide gel electrophoresis and transferred onto polyvinylidene difluoride membranes. Antibodies were applied and incubated as per the manufacturer instructions. The horseradish peroxidase-conjugated secondary antibodies were detected using an enhanced chemiluminescent substrate (Millipore, P90720). The images were captured using an LAS4000 luminescent image analyzer (Fujifilm). Band intensities were quantified using Image J software (NIH).

Primary antibodies against the following were used: LC3 (Novus Biologicals, NB100-2220), ubiquitin (Covance, MMS-258R), and β-tubulin, (Abcam, ab108342), PLD2 (Santa Cruz Biotechnology, sc-25513), PLD1 (Cell Signaling Technology, #3832), p62/SQSTM1(Cell Signaling Technology, #5114), Akt (Cell Signaling Technology, #9272S), phospho-Akt (Ser^473^) (Cell Signaling Technology, #9271S), ULK1(Cell Signaling Technology, #8054), phospho-ULK1 (Ser^555^) (Cell Signaling Technology, #5869), AMPK (Cell Signaling Technology, #2532), phospho-AMPK (Thr^172^) (Cell Signaling Technology, #2535), Beclin1 (Bethyl laboratories, A302-567A), and Atg14L (Sigma Aldrich, A6358).

### RNA isolation and quantitative real time polymerase chain reaction (PCR)

Total RNA from cells and tissues was extracted using TRIzol reagent RNA extraction kit (Life Technologies, 15596026) according to the manufacturer instructions and used to synthesize cDNA, using the TOPscript^TM^ RT DryMIX kit (dT18 plus) (Enzynomics, RT200). Real-time PCR analysis was performed with ABI7300 (Applied Biosystems Incorporation) using TOPreal^TM^ qPCR 2X PreMIX (SYBR Green with high ROX) (Enzynomics, RT501S). To normalize the expression of the genes, cyclophilin A was used. The list of primer sequences is shown in Supplementary Table 1.

### Lipid analyses

To extract ceramide, DAG and PA, 20 mg of liver was homogenized in cold methanol/phosphate-buffered saline (2:1 v/v) for ceramide and cold chloroform/methanol (2:1 v/v) for DAG and PA. Organic phase were separated from aqueous phase by adding chloroform and water. After centrifugation, the organic layer was collected, dried under nitrogen flow, and reconstituted with 0.1% formic acid in methanol for ceramide, hexane/methylene chloride/ether (95/4.5/0.5 v/v/v) for DAG, and methanol for PA. The internal standards for ceramide, DAG, and PA are C17:0 ceramide (500 ng/ml), 1, 3-dipenta decanoin and phosphatidic acid (C10:0/10:0), respectively. Diacylglycerol was separated from triacylglycerol using preconditioned columns (Waters SepPak Cartridge, WAT020845) and eluted with hexane/ethyl acetate (85/15 v/v) under a low negative pressure. Ceramide and DAG were measured by LC-MS/MS 4000 Q TRAP (Applied Biosystems). Phosphatidic acid was analyzed by Agilent 6490 Triple Quadrupole LC/MS System (Agilent Technologies). Anionic phospholipids (PI, cardiolipin, PS and phosphoinositides) were measured using HPLC combined with suppressed conductivity (Dionex), as previously described^54^.

### Metabolic measurements

Blood glucose during hyperinsulinemic-clamp and glucose tolerance test was measured using the GM9 Glucose Analyzer (Analox Instruments). Lipids in tissue were extracted using the method of Bligh and Dyer^55^ and hepatic triglyceride, cholesterol and plasma NEFA were measured by colorimetric assay kits from Wako (Wako Pure Chemical Industries, 279-75401). Plasma triglyceride, total cholesterol, low-density lipoprotein (LDL), high-density lipoprotein (HDL), ALT, and AST were measured using a Cobas c111 analyzer (Roche). Plasma insulin was measured using Mouse Ultrasensitive Insulin ELISA kits (ALPCO, 80-INSMSU-E01).

### Histology

Liver was collected and frozen in optimal cutting temperature embedding medium (Cell Path, KMA-0100-00A). Paraffin-embedded 5 μm thick sections were stained with H&E (Sigma Aldrich, H9627). Frozen liver sections were stained with Oil red O. The images were visualized by phase-contrast microscopy.

### Fluorescent images

Cells were cultured on Lab-Tek II Chamber Slides (Nalgene Nunc International, Rochester, 154534). The Premo^TM^ Autophagy Tandem Sensor RFP-GFP-LC3B Kit (Life Technologies, P36239) was used to monitor autophagy flux. The cells were treated, followed by fixation in a 4% formaldehyde solution. Fluorescent images were obtained using the Laser Scanning Microscope 700 (Carl Zeiss).

### Transmission electron microscopy images

Liver tissues were quickly isolated from anaesthetized mice; they were then immediately fixed with 2.5% glutaraldehyde in 0.1 M phosphate-buffered saline for at least 2 h at 4 °C. The tissues were post-fixed at room temperature with 1% osmium tetroxide for 1.5 h. Tissue sections with a thickness of approximately 70–80 nm were prepared with an ultramicrotome (Leica) and collected on copper grids. The samples were analyzed with a Cryo-Tecnai F20 (Field Electron and Ion).

### Statistical analyses

All data are presented as means ± standard error (SE). The significance of the differences in mean values between the *Pld1*^+/+^ and *Pld1*^−/−^ mice was evaluated using unpaired Student’s *t*-test.

## Additional Information

**How to cite this article:** Hur, J. H. *et al*. Phospholipase D1 deficiency in mice causes nonalcoholic fatty liver disease via an autophagy defect. *Sci. Rep.* **6**, 39170; doi: 10.1038/srep39170 (2016).

**Publisher's note:** Springer Nature remains neutral with regard to jurisdictional claims in published maps and institutional affiliations.

## Supplementary Material

Supplementary Dataset

## Figures and Tables

**Table 1:** Plasma lipid analyses.

Group	WT	KO
	(n = 9)	(n = 11)
FFA (mEq/L)	0.69 ± 0.04	0.74 ± 0.03
TG (mg/dL)	74.1 ± 4.9	64.9 ± 3.0
Total Cholesterol (mg/dL)	159.9 ± 6.4	159.3 ± 4.7
HDL-Chol (mg/dL)	108.8 ± 4.3	112.3 ± 2.4
LDL-Chol (mg/dL)	25.4 ± 1.9	27.6 ± 2.1
ALT (U/L)	47.4 ± 4.5	69.2 ± 7.9*
AST (U/L)	75.3 ± 3.6	92.9 ± 6.7*

^*^P < 0.05, when compared to the *Pld1*^+/+^.
