# Supplementary material for: Phospholipase D1 deficiency in mice causes nonalcoholic fatty liver disease via an autophagy defect
Source: Sci Rep. 2016 Dec 15;6:39170. doi: 10.1038/srep39170 (PMC5156943; doi:10.1038/srep39170)
Supplement: Supplementary Dataset [file srep39170-s1.doc]

**Supplementary Information**

**Phospholipase D1 deficiency in mice causes nonalcoholic fatty liver disease via an autophagy defect**

Jang Ho Hur^1,2^, Shi-Young Park^1,2^, Claudia Dall’Armi^3^, Jae Sung Lee^1,2^, Gilbert Di Paolo^3^, Hui-Young Lee^1,2^, Mee-Sup Yoon^1,2*^, Do Sik Min^4*^, Cheol Soo Choi^1,2,5*^

**Table of Contents**

Supplementary figures…………………………………………………………………2

Supplementary Fig. 1……………………………………………………………….2

Supplementary Fig. 2…………………………………………………………….....3

Supplementary Fig. 3…………………………………………………………….....4

Supplementary Fig. 4……………………………………………...………………..5

Supplementary Fig. 5…………………………………………………………….....6

Supplementary Fig. 6…………………………………………………………….....7

Supplementary Fig. 7…………………………………………………………….....8

Supplementary Tables…………………………………………………………………9

Supplementary table 1……………………………………………………………...9

**Supplementary figures**

**
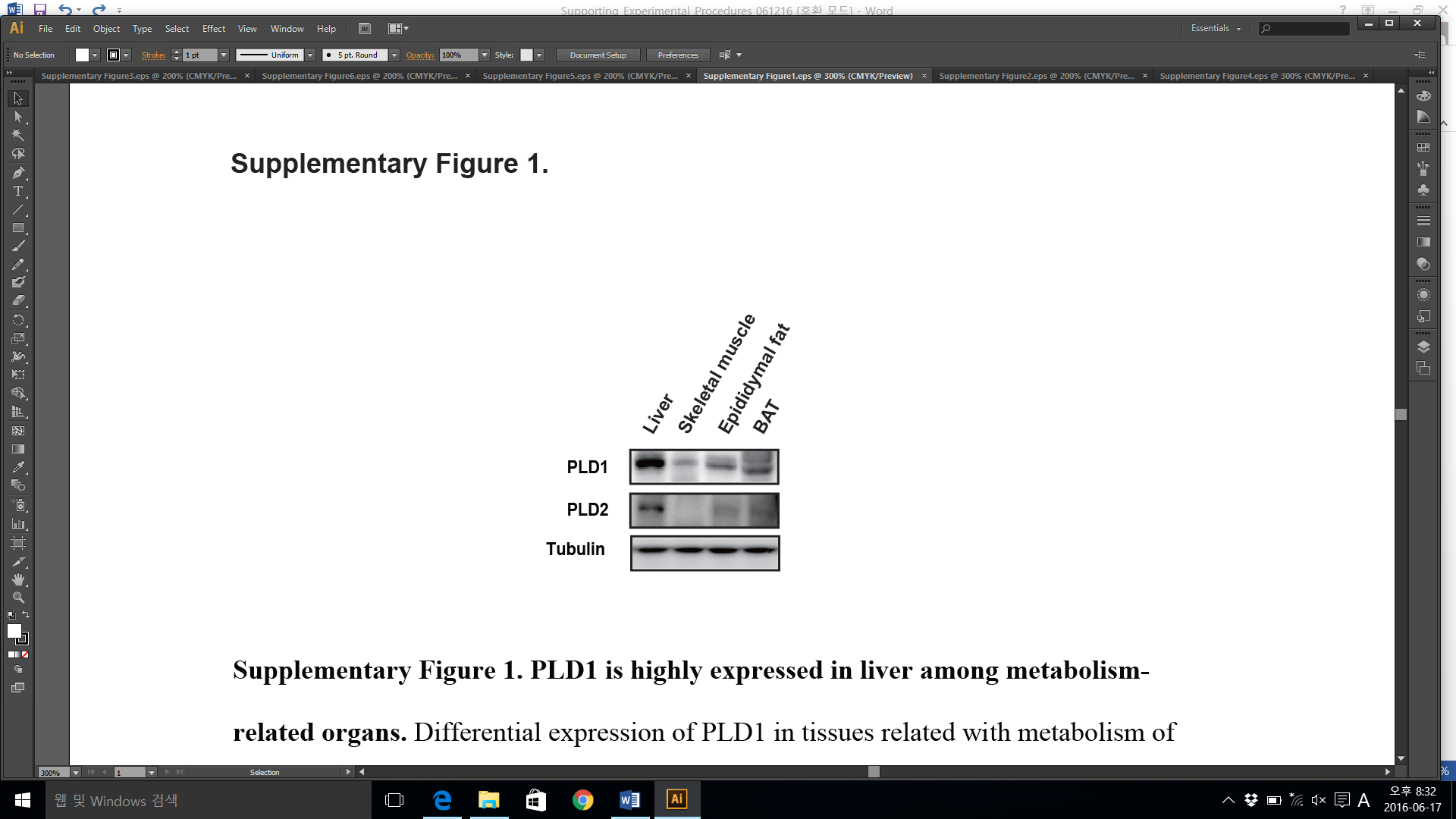
**

**Supplementary Figure 1. PLD1 is expressed more highly in the liver than other metabolism-related organs.** *Pld1^+/+^* mice were fed RC for 13 weeks (n = 10, per group) and tissues were isolated. Tissue lysates were analyzed by western blotting. Representative blots are presented from 3 to 5 independent experiments.


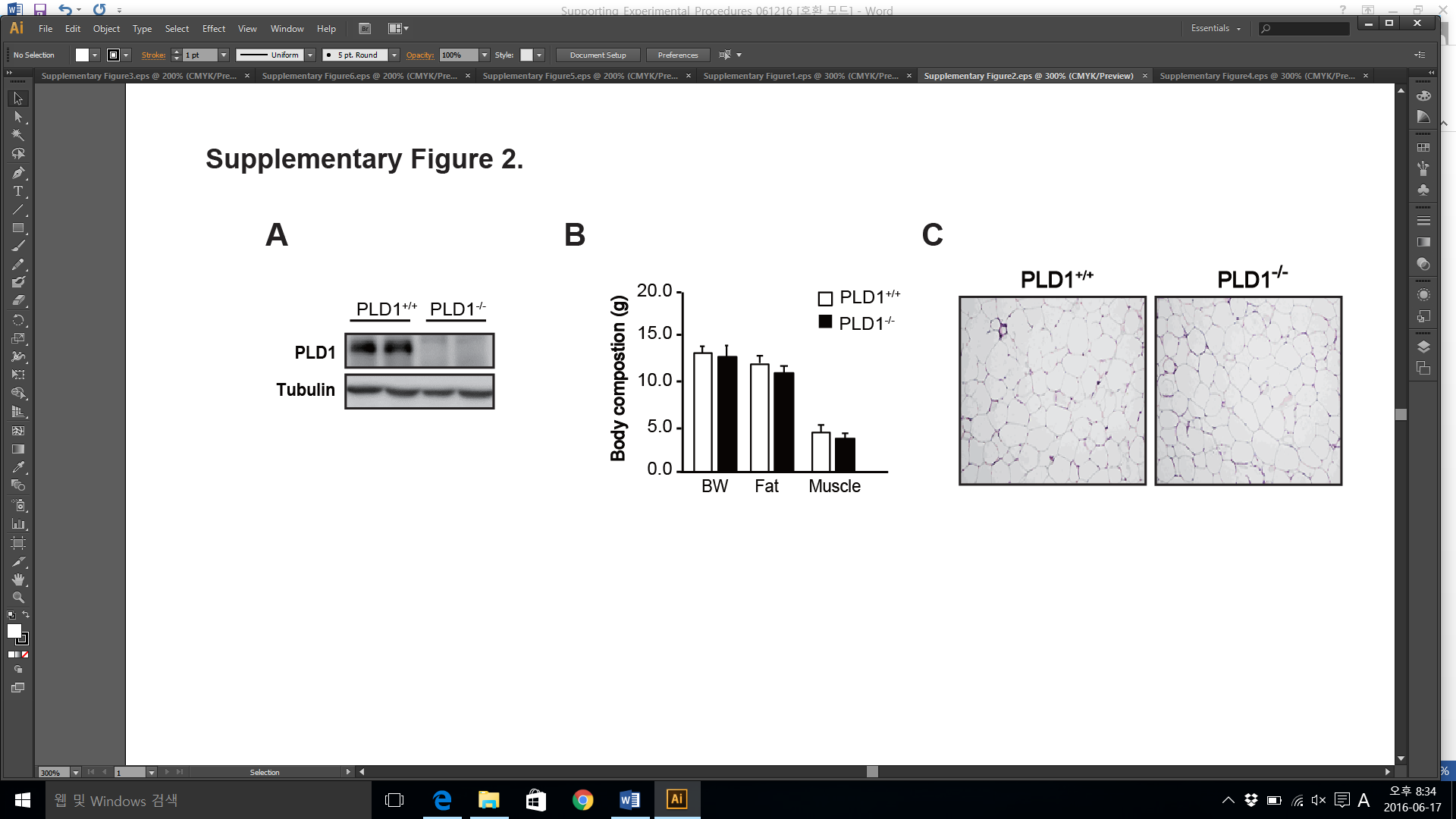


**Supplementary Figure 2. Characterization of *Pld1^-/-^* mice.** *Pld1^+/+^* and *Pld1^-/-^* littermates were fed a HFD for 4 weeks at the age of 13 weeks (n = 9–11 per group). (A) The livers were isolated and analyzed with western blotting. (B) The changes in body weight, fat, and muscle mass were measured by ^1^H magnetic resonance spectroscopy. (C) Epididymal fats were stained with H&E. Representative images were obtained at 100× magnification from 3–5 independent experiments. The data are presented as means ± SE or representative blots from 3 to 5 independent experiments.


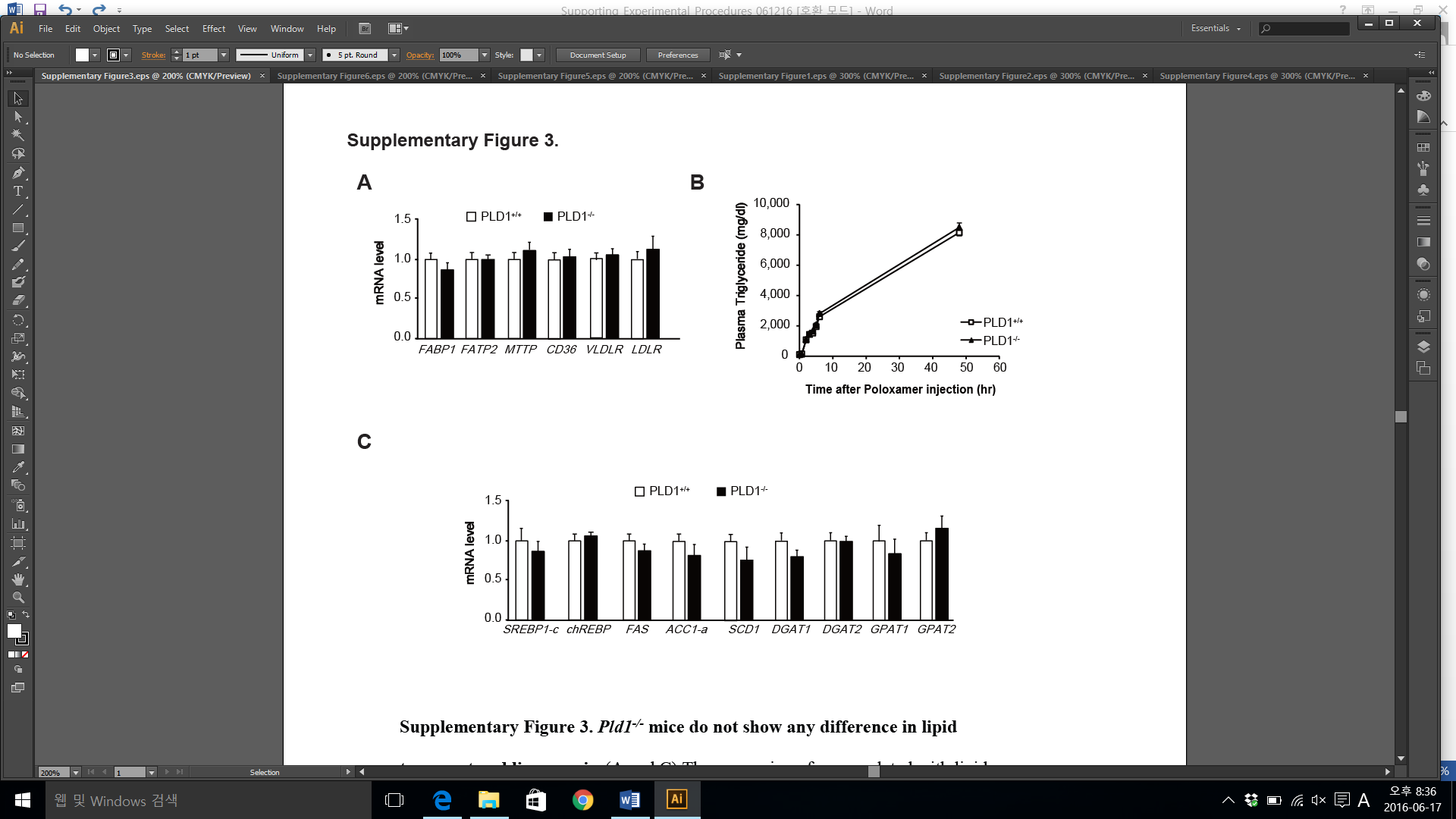


**Supplementary Figure 3. *Pld1^-/-^* mice do not show any differences in lipid transport or lipogenesis.** The *Pld1^+/+^* and *Pld1^-/-^* littermates were fed a HFD for 4 weeks starting at the age of 13 weeks (n = 9–11 per group). The expression levels of genes related to (A) lipid transport and (C) lipogenesis were analyzed using qRT-PCR. (B) The mice were fasted overnight and injected with Poloxamer 407 (1 g/kg). Plasma triacylglycerol levels were measured in *Pld1^+/+^* and *Pld1^-/-^* mice at the indicated time points. The data are presented as means ± SE from 3 to 5 independent experiments.


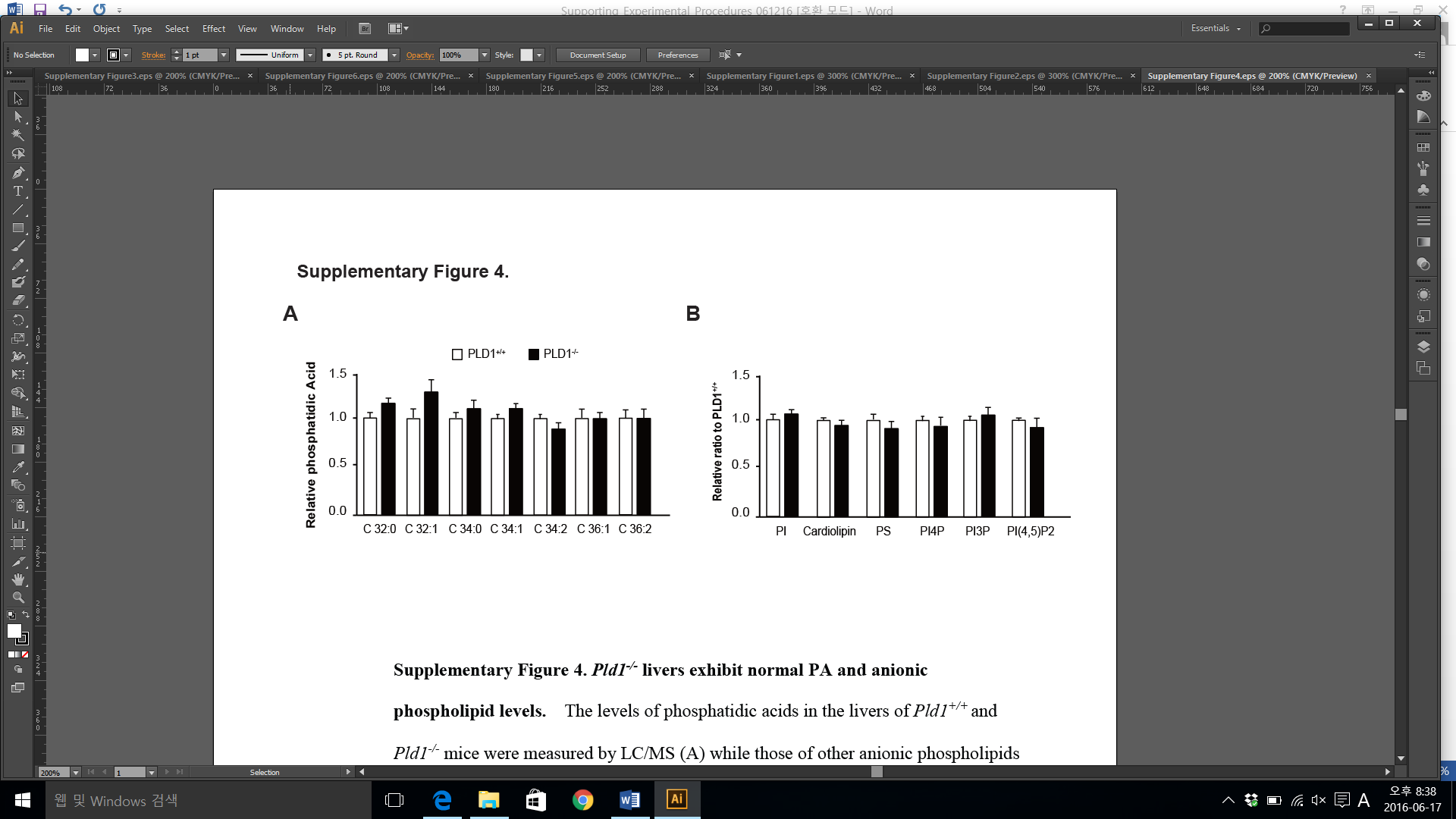


**Supplementary Figure 4. Levels of PA and anionic phospholipids did not differ between *Pld1^-/-^* and *Pld1^+/+^* livers.** The *Pld1^+/+^* and *Pld1^-/-^* mice were fed a HFD for 4 weeks starting at the age of 13 weeks (n = 9–11 per group). The livers were isolated. (A) The levels of phosphatidic acid in the livers of *Pld1^+/+^* and *Pld1^-/-^* mice were measured by LC/MS. (B) The levels of other anionic phospholipids were measured by HPLC combined with suppressed conductivity. N = 3–5 per group, mean ± SE, *P < 0.05.

**
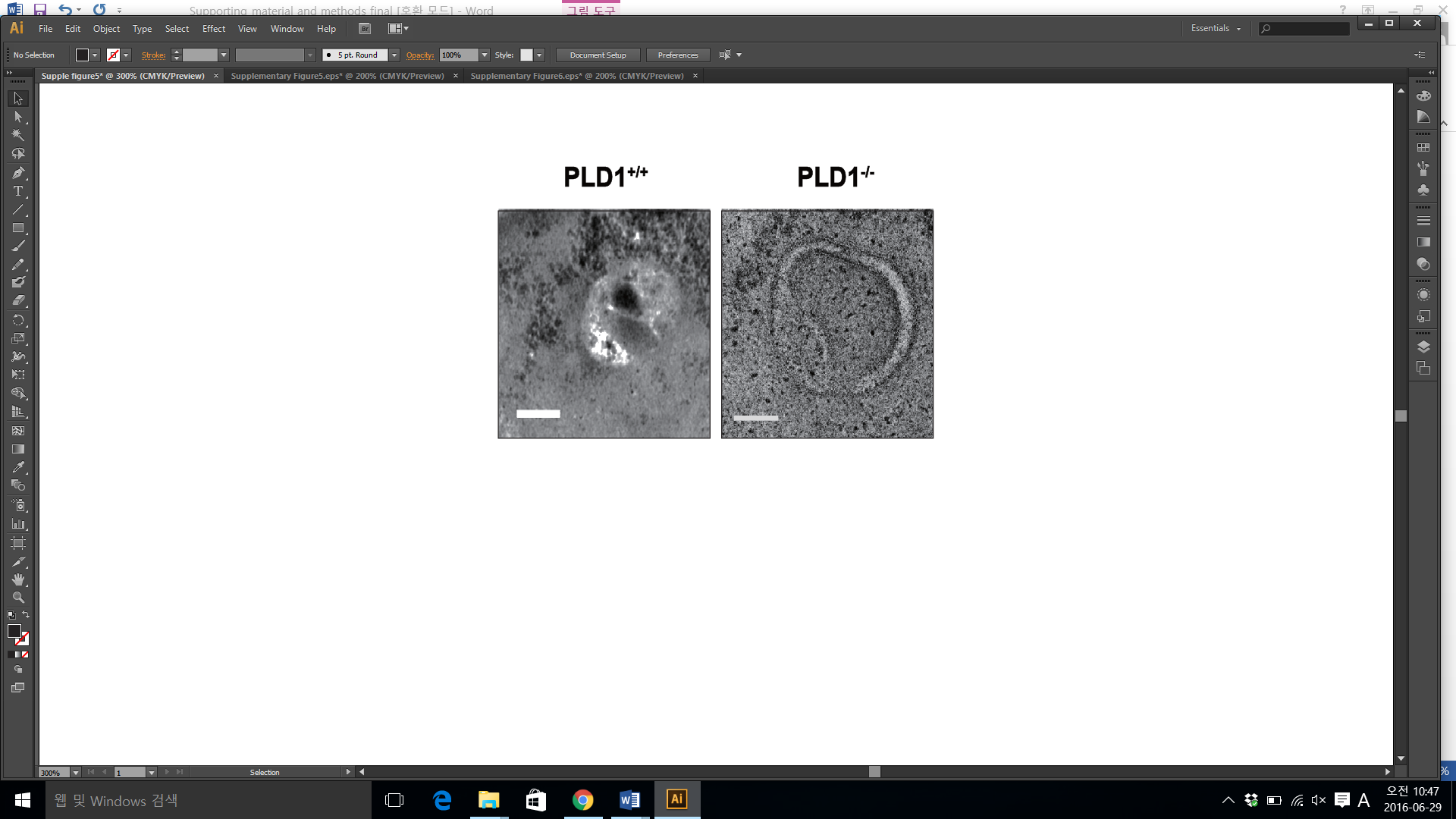
**

**Supplementary Figure 5.** **Autolysosome were significantly reduced in *Pld1^-/-^* mice liver**. **.** Liver were isolated from *Pld1^+/+^* and *Pld1^-/-^* mice were fed a HFD for 4 weeks.

Autophagosome and autolysosome in the livers from *Pld1^+/+^* and *Pld1^-/-^* littermates were visualized by transmission electron microscopy. Based on an electron microscopy analysis of the liver, autolysosomes (dense structures with membranes) were reduced in *Pld1^-/-^* mice and autophagosomes (AP) with lipids (light structures with membranes) were primarily observed in *Pld1^-/-^* mice.


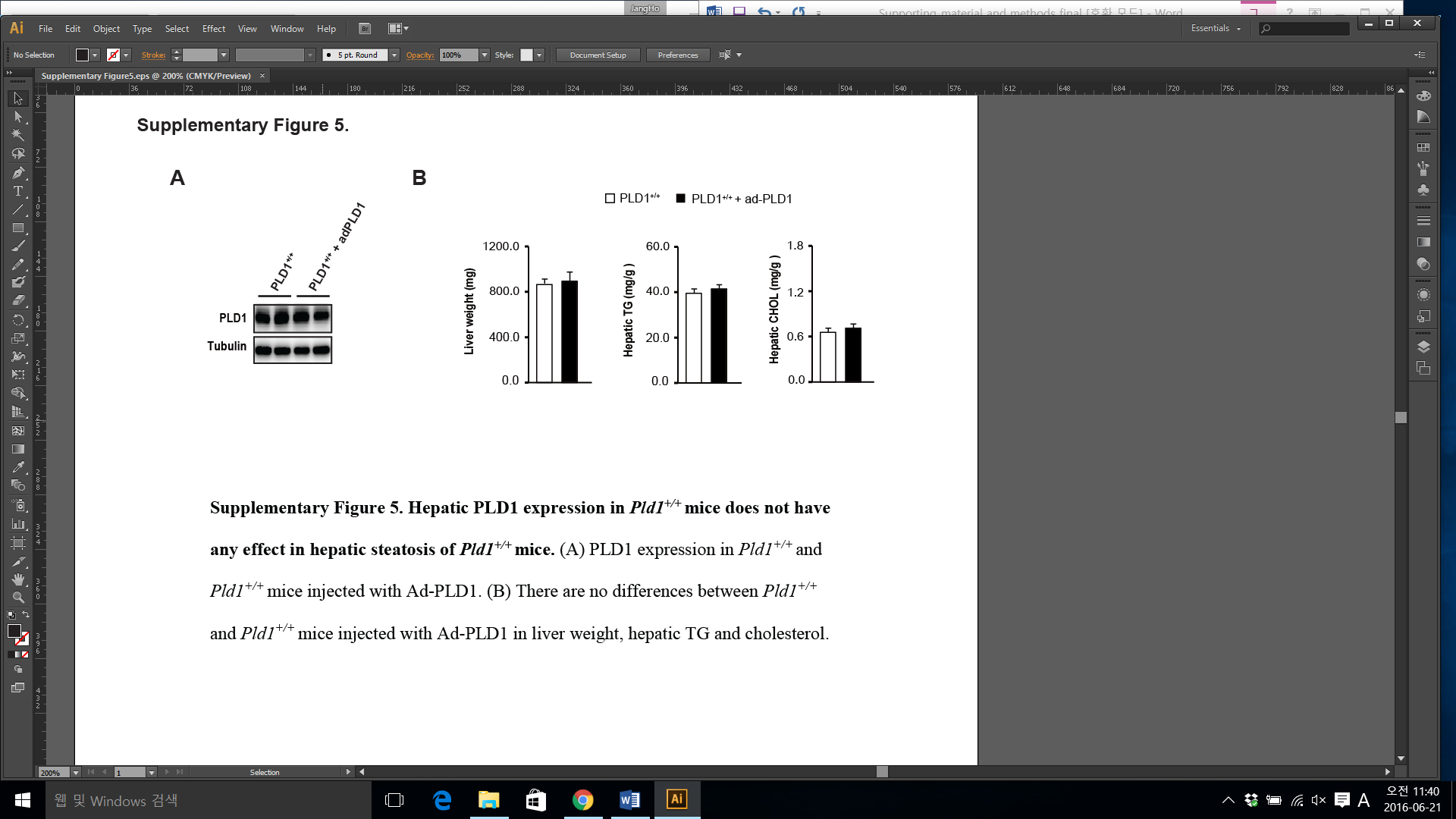


**Supplementary Figure 6.** **Adenoviral gene delivery in *Pld1^+/+^*** **mice does not affect PLD1 levels and hepatic steatosis in *Pld1^+/+^*** **mice.** (A) After feeding the mice with a HFD for one week beginning at 13 weeks, the mice were injected with adGFP or adPld1 (1 × 10^9^ PFU) by tail vein injection. At 12 days post-injection, mice were sacrificed for analyses. Mouse liver lysates were analyzed by western blotting. (B) Mice were maintained as described in (A). The weights of mouse livers were measured and hepatic triglycerides and cholesterol were quantitatively analyzed. The data are presented as means ± SE or representative blots from 3 to 5 independent experiments.


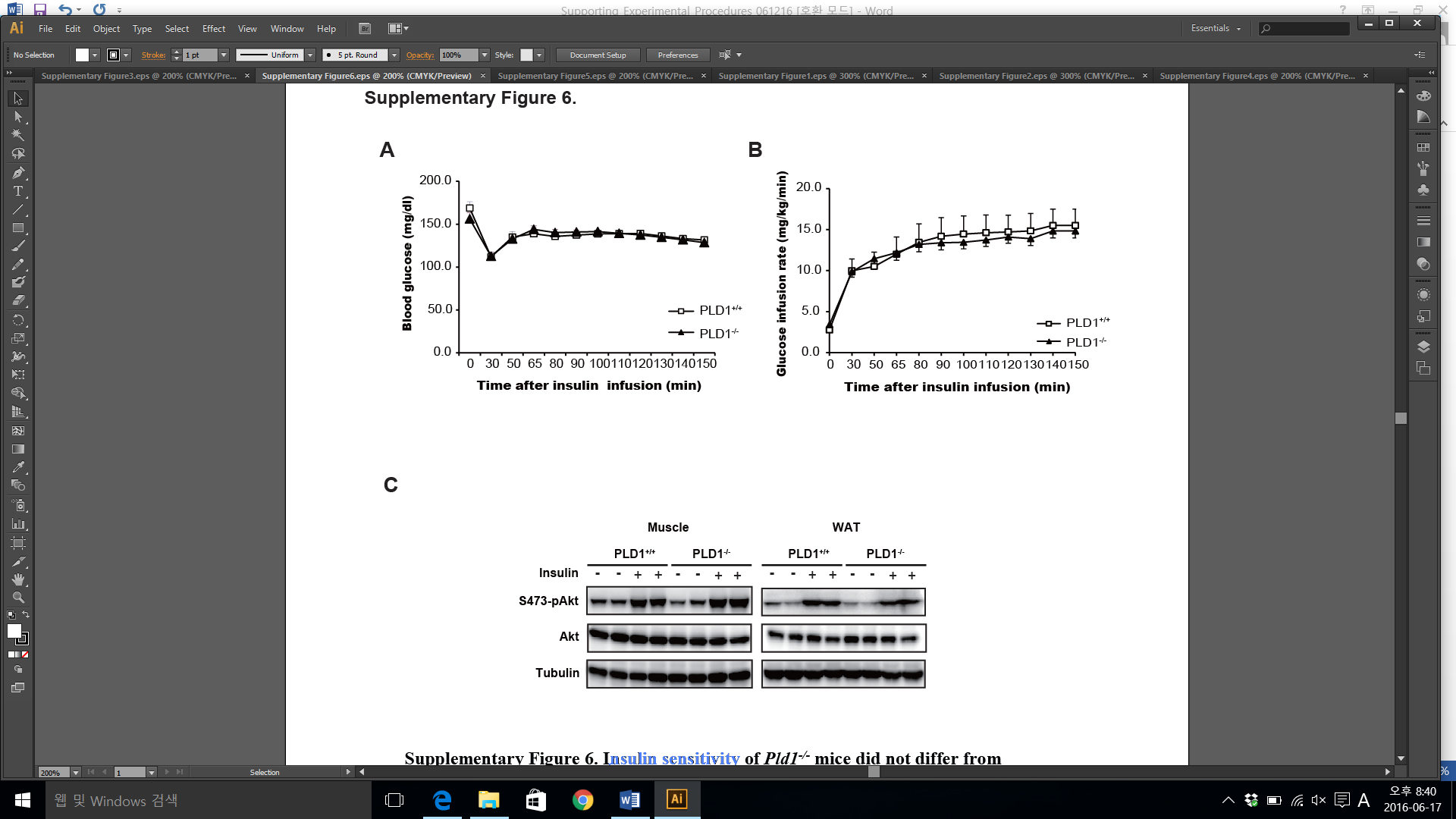


**Supplementary Figure 7.** **Insulin sensitivity of *Pld1^-/-^* mice did not differ from that of *Pld1^+/+^* mice.** After they were fed a HFD for 4 weeks beginning at age 13 weeks, mice were fasted overnight. (A and B) Hyperinsulinemic-euglycemic clamp techniques were used. (A) Blood glucose level and (B) the glucose infusion rate (GINF) of *Pld1^+/+^* and *Pld1^-/-^* mice were measured. (C) Mice were injected with saline or insulin (IP, 1.5 units/kg; n = 5 per group), and sacrificed at 10 min after injection. The lysates of muscle and epididymal fat were analyzed with western blotting. The data are presented as means ± SE or representative blots from 3 to 5 independent experiments. **P* < 0.05 versus *Pld1^+/+^* mice.

**Supplementary table1. Primers used in the study.**

| Gene | Sequence |
| --- | --- |
| *PLD1 F* | 5′ - AGT GCT TCA GAC TTG TCC TGG GTT - 3′ |
| *PLD1 R* | 5′ - TAT GGT AGC GTT TCG AGC TGC TGT - 3′ |
| *PLD2 F* | 5′ - TTG CGG AAG CAC TGT TTC AGT GTC - 3′ |
| *PLD2 R* | 5′ - TTG TTC TCC GCT GTT TCT TGC CAC - 3′ |
| *Cpt1-a F* | 5′ - TGG GCT ACT CAG AGG ATG G - 3′ |
| *Cpt1-a R* | 5′ - AAG GTG TCA AAT GGG AAG G - 3′ |
| *PPAR-α F* | 5′ - AAC TGG ATG ACA GTG ACA TTT CC - 3′ |
| *PPAR-α R* | 5′ - CCC TCC TGC AAC TTC TCA AT - 3′ |
| *Acat1 F* | 5′ - CAG GAA GTA AGA TGC CTG GAA C -3′ |
| *Acat1 R* | 5′ - TTC ACC CCC TTG GAT GAC ATT - 3′ |
| *Acadvl F* | 5′ - CTA CTG TGC TTC AGG GAC AAC - 3′ |
| *Acadvl R* | 5′ - CAA AGG ACT TCG ATT CTG CCC - 3′ |
| *FABP1 F* | 5′ - ATG AAC TTC TCC GGC AAG TAC C - 3′ |
| *FABP1 R* | 5′ - CTG ACA CCC CCT TGA TGT CC - 3′ |
| *FATP2 F* | 5′ - ATT CGT GCC AAG TCT CTG CT - 3′ |
| *FATP2 R* | 5′ - GTA AAA GAC GGA CAC GGC AT - 3′ |
| *MTTP F* | 5′ - CTC TTG GCA GTG CTT TTT CTC T - 3′ |
| *MTTP R* | 5′ - GAG CTT GTA TAG CCG CTC ATT - 3′ |
| *CD36 F* | 5′ - ATT GGT CAA GCC AGC T - 3′ |
| *CD36 R* | 5′ - TGT AGG CTC ATC CAC TAC - 3′ |
| *VLDLR F* | 5′ - GGC AGC AGG CAA TGC AAT G -3′ |
| *VLDLR R* | 5′ - GGG CTC GTC ACT CCA GTC T - 3′ |
| *LDLR F* | 5′ - AGG CTG TGG GCT CCA TAG G - 3′ |
| *LDLR R* | 5′ - TGC GGT CCA GGG TCA TCT - 3′ |
| *SREBP1 F* | 5′ - GTA CCT GCG GGA CAG CTT AG - 3′ |
| *SREBP1 R* | 5′ - TCA GGT CAT GTT GGA AAC CA - 3′ |
| *chREBP F* | 5′ - AGA ACC GAC GTA TCA CAC ACA TCT - 3′ |
| *chREBP R* | 5′ - CAG GGT GTC GAA TCC TAG CTT AA - 3′ |
| *FASN F* | 5′ - CCT GCT ATC ATC TGA CTT CCT CT - 3′ |
| *FASN R* | 5′ - AGG GTG GTT GTT AGA AAG ATC AA - 3′ |
| *ACC1 F* | 5′ - GCG GGA GGA GTT CCT AAT TC - 3′ |
| *ACC1 R* | 5′ - TGT CCC AGA CGT AAG CCT TC - 3′ |
| *SCD1 F* | 5′ - CCG GAG ACC CCT TAG ATC GA -3′ |
| *SCD1 R* | 5′ - TAG CCT GTA AAA GAT TTC TGC A - 3′ |
| *DGAT1 F* | 5′ - TCC GTC CAG GGT GGT AGT G - 3′ |
| *DGAT1 R* | 5′ - TGA ACA AAG AAT CTT GCA GAC GA - 3′ |
| *DGAT2 F* | 5′ - GCG CTA CTT CCG AGA CTA CTT - 3′ |
| *DGAT2 R* | 5′ - GGG CCT TAT GCC AGG AAA CT - 3′ |
| *GPAT1 F* | 5′ - CAA CAC CAT CCC CGA CAT C - 3′ |
| *GPAT1 R* | 5′ - GTG ACC TTC GAT TAT GCG ATC A - 3′ |
| *GPAT2 F* | 5′ - CAC TGC TCC GAG GTT TTG ATG - 3′ |
| *GPAT2 R* | 5′ - AGG TTG GCA GCA ATT CCA TAC - 3′ |
| *Cyclo A F* | 5′ - CAA GAC TGA ATG GCT GGA TG - 3′ |
| *Cyclo A R* | 5′ - TGG TGA TCT TCT TGC TGG TC - 3′ |
